# Non-invasive Brain Stimulation: Probing Intracortical Circuits and Improving Cognition in the Aging Brain

**DOI:** 10.3389/fnagi.2018.00177

**Published:** 2018-06-08

**Authors:** Joyce Gomes-Osman, Aprinda Indahlastari, Peter J. Fried, Danylo L. F. Cabral, Jordyn Rice, Nicole R. Nissim, Serkan Aksu, Molly E. McLaren, Adam J. Woods

**Affiliations:** ^1^Department of Physical Therapy, University of Miami Miller School of Medicine, Miami, FL, United States; ^2^Evelyn F. McKnight Brain Institute, University of Miami Miller School of Medicine, Miami, FL, United States; ^3^Berenson-Allen Center for Noninvasive Brain Stimulation, Division of Cognitive Neurology, Department of Neurology, Beth Israel Deaconess Medical Center, Harvard Medical School, Boston, MA, United States; ^4^Department of Clinical and Health Psychology, Department of Neuroscience, Center for Cognitive Aging and Memory, McKnight Brain Institute, University of Florida, Gainesville, FL, United States

**Keywords:** cognitive aging, non-invasive brain stimulation, TMS, tDCS, cognition, neuromodulation

## Abstract

The impact of cognitive aging on brain function and structure is complex, and the relationship between aging-related structural changes and cognitive function are not fully understood. Physiological and pathological changes to the aging brain are highly variable, making it difficult to estimate a cognitive trajectory with which to monitor the conversion to cognitive decline. Beyond the information on the structural and functional consequences of cognitive aging gained from brain imaging and neuropsychological studies, non-invasive brain stimulation techniques such as transcranial magnetic stimulation (TMS) and transcranial direct current stimulation (tDCS) can enable stimulation of the human brain *in vivo*, offering useful insights into the functional integrity of intracortical circuits using electrophysiology and neuromodulation. TMS measurements can be used to identify and monitor changes in cortical reactivity, the integrity of inhibitory and excitatory intracortical circuits, the mechanisms of long-term potentiation (LTP)/depression-like plasticity and central cholinergic function. Repetitive TMS and tDCS can be used to modulate neuronal excitability and enhance cortical function, and thus offer a potential means to slow or reverse cognitive decline. This review will summarize and critically appraise relevant literature regarding the use of TMS and tDCS to probe cortical areas affected by the aging brain, and as potential therapeutic tools to improve cognitive function in the aging population. Challenges arising from intra-individual differences, limited reproducibility, and methodological differences will be discussed.

## Introduction

### Aging, cognitive aging, and cognitive impairment

Over the next 50 years, the number of adults aged 60 years or older will double, quickly reaching 1.4 billion people worldwide (United Nations, Department of Economic and Social Affairs, Population Division, 2015). The maintenance of cognitive health and concerns regarding memory loss are consistently cited as top concerns by this population (National Association of Area Agencies on Aging, 2015). Therefore, a greater understanding of the processes underlying the aging brain is relevant.

Cognitive aging is the decline in cognitive abilities resulting from physiologic change with age. The cognitive trajectory of cognitive aging is unique to each individual, resulting from inherent differences in the genetic make-up, life experiences, and level of education. The consequences of cognitive aging can range from subtle, even imperceptible, changes that do not impact quality of life, to more severe declines that antedate dementia (Harada et al., 2013). Even in the absence of disease, cognitive aging can result in selective impairment of certain cognitive domains. Episodic memory begins to decline during mid-life (Nyberg et al., 1996), while semantic memory decreases later, as individuals become elders (Nyberg, 2004; Rönnlund et al., 2005). Executive functioning, especially mental flexibility and response inhibition, also show age-dependent decreases (Wecker et al., 2005). Furthermore, psychomotor processing speed slows (Salthouse, 2010), and the ability to focus attention and/or multi-task becomes more difficult (Carlson et al., 1995; Darowski et al., 2008; Salthouse, 2010).

The neurobiological underpinnings of cognitive aging are multifactorial. Cognitive impairments may result from brain atrophy (cortical thinning or decreased gray matter volume), especially to the prefrontal cortex, temporal lobes, and hippocampus (Raz et al., 2004; DeCarli et al., 2005). The frontal lobe, which is the last to mature (around 25 years old) and the first to start thinning with age (Salat et al., 2004), plays an essential role in higher-order cognitive processes and a variety of critical executive functioning processes such as planning, decision-making, problem solving, and working memory (Nissim et al., 2017). Furthermore, demyelination or lesions to white matter tracts (Dong et al., 2015), imbalances in dopamine and serotonin (Mukherjee et al., 2002; Nyberg, 2004), decreased brain-derived neurotrophic factor (Mattson et al., 2004), increased monoamine oxidase, and deposition of amyloid beta (Aβ) are all linked to increased free radicals and dendritic spine loss (Hsieh et al., 2006).

The highly individual trajectories of cognitive aging pose a challenge clinically in that it is difficult to estimate one's cognitive trajectory and predict conversion to more severe clinical states, such as cognitive impairment or dementia. Annually, ~15–20% of adults above 65 years of age will present with mild cognitive impairment (MCI) (Roberts and Knopman, 2013). In addition, more than 30% of individuals with MCI will develop Alzheimer's disease (AD) (Ward et al., 2013) or other types of dementia (Mitchell and Shiri-Feshki, 2009) within 5 years.

Great strides have been made in the study of the consequences of cognitive aging at the macroscopic level in humans. With the advent of neuroimaging techniques, preclinical pathophysiological changes have been identified as early as 10–15 year before individuals exhibit any cognitive changes (Sperling et al., 2011). In the currently accepted biomarker model of the preclinical stage of AD, evidence of Aβ accumulation and synaptic dysfunction are the first preclinical features observed through cerebrospinal fluid measurements, positron emission tomography and functional magnetic resonance imaging (fMRI) (Sperling et al., 2011).

Non-invasive brain stimulation (NIBS) modalities, which permit direct or indirect electrical stimulation of the human brain *in-vivo*, can be useful as adjunct to other neuroimaging tools in the study of cognitive aging and impairment. NIBS techniques, including transcranial magnetic stimulation (TMS) and transcranial direct current stimulation (tDCS), can be used to assess the functional integrity of intracortical circuits during synaptic dysfunction. Additionally, previous studies have shown the potential therapeutic roles of NIBS to reestablish or normalize activity and metabolism in brain areas affected by aging. The present review will focus on the available literature regarding the use of NIBS in the form of TMS and tDCS in the study of cognitive aging and cognitive decline.

## Transcranial magnetic stimulation (TMS)

TMS is a means of indirect electrical stimulation via electromagnetic induction. Briefly, to generate a single TMS pulse, an electric current is passed through a copper coil generating a magnetic field that is perpendicular to the plane of the coil (Kobayashi and Pascual-Leone, 2003). The rapid rate of change of the magnetic field induces a secondary electric field in the underlying brain tissue (Kobayashi and Pascual-Leone, 2003) with the potential to depolarize neuronal membranes.

With the TMS coil placed on the subject's scalp overlying the motor cortex, by increasing the stimulus output, it is possible to alter the rate of change of the magnetic field so that the induced electrical current can depolarize layer-V pyramidal neurons directly, as well as indirectly through trans-synaptic activation of nearby interneurons (Kobayashi and Pascual-Leone, 2003). The depolarization of the pyramidal neuron propagates along the corticospinal pathway, and elicits a motor evoked potential (MEP) if the firing threshold is sufficient to trigger this process in a population of pyramidal neurons. The amplitude and latency of MEPs induced by the single pulse can be recorded with surface electrodes placed on the muscles of interest, such as the contralateral first dorsal interosseous (FDI) or abductor pollicis brevis (APB). TMS incorporates a variety of techniques. Single-pulse and paired-pulse TMS comprise paradigms that enable the probing of the cortical reactivity and functional integrity of excitatory and inhibitory intracortical circuits. Repetitive TMS protocols have traditionally been employed for therapeutic use, but also enable an assessment of mechanisms of plasticity.

### Single-pulse TMS

Single-pulse TMS is the building block of all TMS protocols, but on its own, can be used to probe the reactivity of the motor cortex (as well as non-motor regions if electroencephalography is used concurrently). The most ubiquitous measure in TMS is the motor threshold (MT). The MT is defined as the percentage of the maximum stimulator output required to elicit MEPs of a minimum amplitude in at least 50% of consecutive trials [typically 50 μV at rest (resting MT; rMT), or 200 μV during ~20% of maximum contraction (active MT; aMT); (Rossi et al., 2009)]. The most current recommendations are that the MT be drawn from the number of MEPs acquired over 20 responses (i.e., the intensity that produces MEPs in 10/20 trials), to increase reliability (Rossini et al., 2015).

Locally, the MT assesses the reactivity of the motor pathway from the motor cortex, along the corticospinal tract, and peripheral muscle. Globally, the MT is influenced by whole-brain structural changes that alter the scalp-to-cortex distance, as they alter the amount of energy required to bring corticospinal neurons to threshold. Stokes et al. (2007) assessed the influence of increasing coil-to-cortex distance utilizing acrylic separators, and found that for every millimeter, there was an increase in ~2.8% in the MT. Given that age-related atrophy of cortical tissue and increased ventricular volume can increase the coil-to-cortex distance, the MT is a relevant measure to explore in the aging brain. Another metric of overall corticomotor excitability obtained using TMS single pulses is the input-output (IO; also stimulus-response or recruitment) curve. The IO curve evaluates changes in MEP intensity in response to systematically varying TMS intensity and can be influenced by the patterns of recruitment and synchronization of neurons that contribute to the corticospinal volley (Devanne et al., 1997; Chen et al., 1998). This stimulus-response relationship is best determined with a full range of stimulus intensities from sub-threshold (null responses) to a saturation plateau (where further increases in intensity do not elicit larger MEPs). The plotted curve is typically sigmoidal in nature and can be successfully fitted using a Boltzmann function (Devanne et al., 1997).

The IO curve gives rise to a number of distinct parameters can be used to evaluate cortico-motor excitability. Among these the most common are: MEP_MAX_ (the largest MEP that can be elicited, corresponding to the plateau); S_50_ (the stimulation intensity that produces an MEP corresponding to 50% of the maximum); the maximal slope (corresponding to the steepest part of the curve), and the x-intercept (the stimulus intensity where the tangent of slope crosses) (Devanne et al., 1997; Ridding and Rothwell, 1997; Carroll et al., 2001). The advantage of using the input-output curves is that each parameter can be used as an outcome measure, providing complementary information about the excitability at a specific levels of excitability of the corticospinal pathway (Kukke et al., 2014). One challenge for collecting IO curves is being able to stimulate at high enough intensities to reach the plateau. This is especially pertinent for aging given the impact of cortical atrophy on coil-to-cortex distance discussed above. An alternative approach is to normalize MEP responses from TMS to the maximum M-wave that can be elicited by peripheral electrical stimulation.

### Single-pulse TMS in cognitive aging and impairment

While there is evidence for increased rMT in older adults (McGinley et al., 2010; Young-Bernier et al., 2012), some studies also found rMT to be unchanged when compared to young adults (Peinemann et al., 2001; Oliviero et al., 2006; Opie and Semmler, 2014). This discrepancy may be explained by individual patterns of cortical atrophy that are difficult to account for in a cross-sectional analysis unless coil-to-cortex distance can be individually assessed with structural magnetic resonance imaging (MRI).

Another interpretation for the lack of agreement in the age-related differences in rMT is the possibility that the MT goes through different stages during the transition from young adulthood to cognitive aging. In support of this idea, Shibuya et al. (2016) assessed the rMT in 113 individuals with ages ranging from 20 to 83 years of age, making comparisons between subgroups in each decade (Shibuya et al., 2016). The pattern of age-related changes in rMT followed a quadratic curve, with significant differences in each decade. Beginning at 20 years, the rMT increased until age 50, slowly decreasing after that (Shibuya et al., 2016). Ultimately, longitudinal studies are needed to confirm this hypothesis.

Beyond rMT, there is evidence that the stimulus-response relationship may itself be altered in the aging brain. Pitcher et al. (2003) compared the input-output curves of young adults and older adults. The older cohort demonstrated a rightward shift in the x-intercept, wherein higher intensities were necessary to achieve the maximum MEP, while the slope and rMT were not different (Pitcher et al., 2003). The implication of these results is that common approaches for evaluating cortical excitability, such as measuring the intensity necessary to achieve an MEP of 1 mV or averaging the amplitude of MEPs at a specific intensity relative to the rMT, may be misleading because they would represent excitability at different points on the input-output curves for each subject. Thus, future studies should consider incorporating input-output curves into their neurophysiologic assessments in older adults.

In individuals with cognitive impairment due to AD or vascular dementia, the majority of studies have found evidence of cortical hyperexcitability, evidenced by a reduction of rMT when compared with their cognitively intact peers (Carvalho et al., 1997; Di Lazzaro et al., 2002, 2004; Pennisi et al., 2011). However, one study did not find differences in MT between older healthy adults, individuals with early-onset dementia and individuals with fronto-temporal dementia (Pierantozzi et al., 2004), attributing their results to having a homogeneous sample with strict age and cognition-matched controls, though failure to control for coil-to-cortex distance cannot be ruled out.

It may seem counterintuitive that individuals with cognitive impairment would have reduced rMT, since they are more likely to have atrophy (and thus increased cortex-to-coil distance, which would *increase* rMT). This finding might be partly explained by metabolic changes in the neurotransmitter systems that regulate resting membrane potential. There is evidence for a disruption in the availability of neurotransmitters such as gamma-Aminobutyric acid (GABA), glutamate and acetylcholine (Ferreri et al., 2003; Di Lazzaro et al., 2004; Koliatsos et al., 2006) that are consistent with the increase in cortical excitability (and a reduction in the rMT) in AD. Furthermore, there is increasing evidence that hyperexcitability (as indexed by rMT) is related to the severity of cognitive dysfunction in AD (Khedr et al., 2011). Future studies should seek to fully address the relationship between changes in neurotransmitters and rMT in individuals with cognitive impairment.

Taken together, longitudinal measurements of the MT may be more useful to capture the electrophysiological correlates of the gray matter volume loss and degeneration of white matter associated with the aging brain than cross-sectional measurements. Furthermore, few studies report structural or cognitive performance data in the participants. Given the variability in trajectories in cognitive aging, it would be desirable to collect data on the baseline neuropsychological performance and structural integrity of cortical areas, to gain a better understanding of the electrophysiological implications of cognitive and volumetric changes in cognitive aging.

Despite the high variability in cross-sectional measurements of rMT, there is high test-retest reliability for rMT measurements in healthy older adults [intraclass correlation coefficient (ICC) of 94; (Christie et al., 2007)]. This finding has been replicated with the further finding that reproducibility (as measured by Cronbach's α) was higher with using monophasic TMS pulses (α = 0.94) then biphasic TMS pulses (α = 0.83) (Fried et al., 2017). In individuals with AD, reproducibility of the rMT is high and equivalent with both monophasic and biphasic TMS (α = 0.98). The aMT is more variable (α = 0.77 for healthy older adults and 0.85 for individuals with AD) (Fried et al., 2017). While there may be changes in the MT throughout the lifespan, the high reproducibility of this measure within an individual may be useful to demonstrate such transitions.

While the test-retest reliability for measurements of the input-output curve has not been performed in older adults, there is evidence on the test-retest reliability of MEPs along different points of the input-output curve in young adults (Brown et al., 2017). The ICC was highest at 130% rMT (0.70), and decreased at higher levels of intensity (0.68 at 150% rMT, and 0.64 at 175% rMT) (Brown et al., 2017). Future studies should investigate the reliability of input-output curves in older adults.

### Paired-pulse TMS techniques

Paired-pulse TMS refers to the delivery of two TMS pulses in close succession, wherein the first, or conditioning pulse (CP) influences the second, suprathreshold test pulse (TP) (Kobayashi and Pascual-Leone, 2003). Depending on the intensity of the CP and inter-pulse interval, a conditioned TP will result in an MEP of higher or lower amplitude when compared with an unconditioned TP. Different paired-pulse protocols have been developed to probe excitatory and inhibitory intracortical circuits. Short-interval intracortical inhibition (SICI), intracortical facilitation (ICF) and long-interval intracortical inhibition (LICI) are paired-pulse paradigms that use cortical TMS as the CP, whereas short-afferent inhibition (SAI) uses peripheral electrical stimulation as the CP (Kujirai et al., 1993; Tokimura et al., 2000).

### SICI, ICF, and LICI

Short-interval intracortical inhibition (SICI) is assessed when a subthreshold CP (typically delivered at 70–80% rMT) is delivered 1–6 ms prior to the TP (Kujirai et al., 1993; Sanger et al., 2001). There is evidence to support that SICI reflects a form of GABA-a receptor mediated inhibition (Kujirai et al., 1993). Using the same intensity of CP and TP and a slightly longer inter-stimulus interval (ISI) (8–30 ms), one is able to probe intracortical circuits that are associated with facilitation of MEP responses (intracortical facilitation [ICF]) (Kujirai et al., 1993; Sanger et al., 2001). Although the exact mechanisms responsible ICF are not fully understood, it is considered a net-facilitation that is associated with an increased N-methyl-D-aspartate (NMDA) receptor mediated facilitation, combined with a weaker GABA-a-receptor mediated inhibition (Hanajima et al., 1998). When both CP and TP are delivered at suprathreshold intensity (typically 110–130% rMT), and separated by 50–200 ms, a GABA-b receptor mediated inhibition can be observed, termed LICI (Kujirai et al., 1993; Werhahn et al., 1999; Sanger et al., 2001).

### SAI

Short-afferent inhibition (SAI) is a different type of paired-pulse paradigm wherein a peripheral electrical stimulation serves as the CP with a TMS pulse as a TP. In SAI, short (200 μs) electrical currents are applied to the median nerve (above the perceptual sensory threshold) followed 18–50 ms by a suprathreshold TMS pulse over the homologous region of primary motor cortex (M1) (Tokimura et al., 2000). SAI has been attributed *at least partly* to a cholinergic mechanism (Di Lazzaro et al., 2002), and thus, has been utilized in studies assessing the functional integrity of cholinergic circuits in the aging brain. Future studies investigating the mechanisms underlying SAI are needed.

### Paired-pulse TMS techniques in cognitive aging and impairment

The evidence examining paired-pulse measures in cognitive aging and cognitive impairment has produced mixed results. SICI in older adults has been shown to be decreased (Peinemann et al., 2001; Marneweck et al., 2011), increased (McGinley et al., 2010), and unaltered (Oliviero et al., 2006; Opie and Semmler, 2014). SICI was shown to be non-significantly reduced in individuals with AD (Di Lazzaro et al., 2002), but decreased in individuals with early-onset AD (Pierantozzi et al., 2004) and individuals with FTD (Benussi et al., 2017). ICF was found to be both unaltered (Peinemann et al., 2001; Shibuya et al., 2016) and decreased (McGinley et al., 2010) in older adults, unaltered in individuals with AD and decreased in individuals with FTD (Benussi et al., 2017). LICI was found to be both increased (McGinley et al., 2010) and decreased (Opie and Semmler, 2014) in older adults, decreased in individuals with FTD and both decreased (Brem et al., 2013) and unaltered in individuals with AD (Benussi et al., 2017).

Inconsistencies in the effects of paired-pulse protocols across studies can be attributed to several factors. One important factor may be the selection of ISI. Each paired-pulse protocol is associated with a relatively wide range of possible ISIs and the optimal ISI may be different across individuals or between normal and pathological states of cognitive aging. While the majority of TMS paired-pulse studies utilized various ISI's (Peinemann et al., 2001; Di Lazzaro et al., 2002; Pierantozzi et al., 2004; Oliviero et al., 2006; Shibuya et al., 2016; Benussi et al., 2017), others employed only a single ISI for all subjects (McGinley et al., 2010; Marneweck et al., 2011; Opie and Semmler, 2014). Since different individuals will likely show optimal responses at different ISIs, the use of paired-pulse curves (in which the impact of the CP is plotted against the ISI) may decrease the variability and improve the outcomes of future studies.

Another important factor contributing to the variability of paired-pulse protocols is the choice of stimulus intensity for the CP. For SICI and ICF the following CP's were used: 70% rMT (Marneweck et al., 2011; Shibuya et al., 2016); 75% rMT (Peinemann et al., 2001); 80% MT during an active contraction (Opie and Semmler, 2014); 95% rMT (Di Lazzaro et al., 2002; Oliviero et al., 2006; McGinley et al., 2010). In studies that assessed LICI, the conditioning stimulus was given at an intensity that would elicit MEPs in the magnitude of 0.5–1 mV (McGinley et al., 2010) and 120% RMT (Opie and Semmler, 2014). Future studies should standardize the methodology in paired-pulse studies, as variability in methodology introduces unnecessary bias in the comparison across studies.

In addition to the technical aspects mentioned above, some variability in paired-pulse effects may be due to heterogeneity in the neurocognitive status of the control group. Of all studies that assessed paired-pulse techniques, only half (Di Lazzaro et al., 2002; Pierantozzi et al., 2004; Silbert et al., 2006; Marneweck et al., 2011; Pennisi et al., 2011; Young-Bernier et al., 2012, 2014) employed screening of cognitive function for older healthy adults. In addition, two of these (Young-Bernier et al., 2012, 2014) included individuals outside the normative values on the Montreal Cognitive Assessment, but who were classified as healthy on the basis other clinical determinants. The remaining studies did not report if they screened older healthy adults for cognitive impairment (Carvalho et al., 1997; Peinemann et al., 2001; Di Lazzaro et al., 2004; Oliviero et al., 2006; McGinley et al., 2010; Opie and Semmler, 2014; Shibuya et al., 2016).

Unlike paired-pulse TMS, SAI has more consistently been shown to be altered in the aging brain. Two studies found decreased SAI in older adults (Young-Bernier et al., 2012, 2014), but one study found that SAI was unaltered in the comparison between older and young adults (Oliviero et al., 2006). Four studies (Di Lazzaro et al., 2002, 2004; Young-Bernier et al., 2014; Benussi et al., 2017) have reported that individuals with mild to moderate AD demonstrated impaired SAI. Further, one study found that SAI measures were associated with age, independent of the diagnosis of AD or age of onset (Di Lorenzo et al., 2016).

Part of the consistency exhibited in SAI when compared with the TMS paired-pulse paradigms could be explained by the fact that all but one study (Young-Bernier et al., 2014) used various ISI's to characterize SAI. In addition, four (Di Lazzaro et al., 2002, 2004; Di Lorenzo et al., 2016; Benussi et al., 2017) of these six studies normalized the CP to each individual by standardizing it to the N20 component latency of the somatosensory evoked potential of the median nerve. These procedures decrease the influence of inter-individual factors on the overall response. Another potential factor is that central cholinergic intracortical circuits show reliable decays with aging and are primarily implicated in the pathogenesis of AD (Bhandari et al., 2016; Kandimalla and Reddy, 2017). While glutamatergic circuits are also implicated (Kandimalla and Reddy, 2017), these circuits are only partially probed with ICF. Lastly, the precise roles of GABAergic circuits, probed by SICI and LICI in aging and cognitive impairment, are less clear (Bhandari et al., 2016; Kandimalla and Reddy, 2017).

Finally, it is worth noting that many of the studies using paired-pulse techniques may simply be underpowered to reliably detect significant difference between groups given the inter-individual variability in their effects. To this effect, the reproducibility of paired-pulse TMS measures was examined in older healthy adults and individuals with AD (Fried et al., 2017). In the healthy group, reproducibility was moderate for SICI (Cronbach's α = 0.68), low to none for ICF (α = 0.11) and high for LICI (α = 0.98), likely due to floor effects from near complete inhibition. By comparison, all three measures were highly reproducible in the AD group (α ≥0.81) (Fried et al., 2017). The reproducibility of SAI in middle aged adults was moderate (ICC = 0.65–0.67; Brown et al., 2017), but has not been assessed in older adults or those with pathological cognitive aging. Future studies can use these cohort-specific measures of reliability to adjust effect and sample size calculations (Fried et al., 2017).

## Repetitive TMS (rTMS)

Repetitive TMS (rTMS) involves the delivery “trains” of repeated TMS pulses at a set intensity and frequency to a given cortical area (Kobayashi and Pascual-Leone, 2003). rTMS differs from the approaches discussed above in that it offers the possibility of modulating the activity of the stimulated area for a period that outlasts the stimulation application (Kobayashi and Pascual-Leone, 2003). Many factors influence the aftereffects of rTMS, including the frequency, pattern, intensity, and duration of the stimulation. In addition, the basal or ongoing level of activity within the targeted area and associated networks (i.e., state-dependency) can influence the outcome of rTMS (Silvanto et al., 2007).

Conventional rTMS is characterized by the delivery of individual pulses at regular intervals (Kobayashi and Pascual-Leone, 2003). Knowledge of the neurophysiology of rTMS comes primarily from studying its effects in M1. In neurotypical individuals under normal conditions, on-off patterns of high-frequency rTMS (10–20 Hz) tend to increase cortico-motor (i.e., lower rMT and/or higher MEP amplitude), while continuous low frequency rTMS (~1 Hz) tends to reduce excitability (Kobayashi and Pascual-Leone, 2003). In clinical settings (e.g., in the case of medication-resistant major depression) rTMS is used therapeutically with the aim of normalizing aberrant activity in the targeted site and associated networks.

More recently, researchers and clinicians have explored more complex patterned stimulation consisting of *bursts of pulses* at regular intervals (Oberman and Pascual-Leone, 2013). The most commonly used patterned rTMS protocol is Theta-burst stimulation (TBS) (Huang et al., 2005). Modeled after classical protocols for inducing plasticity in the cortex and hippocampal formation, TBS consists of the delivery of a low intensity 50 Hz burst triplet (three pulses with 20 ms inter-pulse interval) repeated at 5 Hz (Huang et al., 2005). TBS is typically applied in one of two patterns: continuous TBS (cTBS), in which the bursts are delivered continuously, has been shown to reduce MEP amplitudes, while intermittent TBS (iTBS), in which the bursts are delivered in 2-s trains with an 8-s delay, has been shown to increase MEP amplitude (Huang et al., 2005). When applied for 600 pulses, cTBS and iTBS have been shown to induce modulation of MEP amplitude for up to 45 min, creating “plasticity curves” that resemble long-term depression (LTD) and long-term potentiation (LTP), respectively in terms of its biochemistry and temporal profile of the effects (Huang et al., 2005). Thus there is a growing interest in the application of TBS to assess the mechanisms of cortical plasticity.

Evidence from human and rodent pharmacology, neuroimaging, and biochemistry studies have shown that the aftereffects of iTBS and cTBS are NMDA receptor-dependent (Huang et al., 2007) and reflect changes in the activity of GABAergic synapses on layer-V pyramidal cells (Stagg et al., 2009b; Funke and Benali, 2011). Huang et al. (2007) found that memantine, a NMDA receptor antagonist, abolished the aftereffects of both iTBS and cTBS, providing evidence that glutamatergic synapses are required for TBS aftereffects. Using magnetic resonance spectroscopy, cTBS was shown to increase GABA metabolism in the stimulated region. Using magnetic resonance spectroscopy, cTBS was shown to increase GABA metabolism in the stimulated region (Stagg et al., 2009b).

Studies of calcium-binding proteins in rodents have shown iTBS likely achieves facilitation by reducing parvalbumin-mediated inhibition of the pyramidal cell output, while cTBS increases inhibition of the pyramidal cell likely by reducing calbindin expression that affects dendritic integration (Funke and Benali, 2011). Complementary evidence from invasive epidural recordings in humans demonstrated iTBS modulates layer-V pyramidal cell activity via monosynaptic connections (Di Lazzaro et al., 2005), while cTBS activates a more complex, trans-synaptic network of interneurons acting on distal dendrites of layer-V pyramidal cells (Di Lazzaro et al., 2008).

The net-effect on the cortical excitability following each stimulation paradigm has been linked to differing patterns of calcium entry in the post-synaptic cell that resemble the patterns of LTP and LTD observed in classical studies of plasticity induction in the trisynaptic pathway of the hippocampus (Huang et al., 2011). In iTBS, interleaving two-second intervals of high-frequency stimulation with 8 s of rest is consistent with a model of stable *high rate* of intracellular calcium in the post-synaptic cell observed in traditional LTP experiments (Huang et al., 2011). By comparison, the uninterrupted high-frequency stimulation of cTBS is associated with an initial rise and eventual *decay in the rate* of calcium entry, leading to an increased overall level of calcium consistent with traditional LTD experiments (Huang et al., 2011). Given the similarities of iTBS and cTBS to LTP and LTD, respectively, the impact of these protocols on cortico-motor excitability is increasingly used to assess the efficacy of neuroplastic mechanisms in relation to aging, disease, and therapeutic interventions (Freitas et al., 2011; Fried et al., 2016, 2017; Gomes-Osman et al., 2017). In addition, given the fact that it leads to modulation of neural activity beyond the stimulation period, TBS has also been employed as a cognitive therapeutic tool.

### TBS and rTMS to assess plasticity in cognitive aging and impairment

Freitas et al. (2011) was the first study to use TBS to gain insights into the impact of normal aging on the mechanisms of plasticity. The authors applied cTBS to M1 in 36 individuals across a wide age range (18–81 y). The authors found negative correlations between age and both the magnitude and duration of cTBS aftereffects, suggesting that LTD-like plasticity is progressively reduced with increasing age. To confirm these findings and further investigate the impact of age, future studies should be performed with sufficient sample sizes to allow for comparisons between age ranges (i.e., per decade or quartile) and ultimately follow the same subjects as they transition from middle to old age.

There have been several additional studies that used TBS to investigate differences in the mechanisms of plasticity between normal and pathological aging, and their behavioral consequences. Fried et al. (2016) compared the response of iTBS to M1 in 24 cognitively intact older adults with type-2 diabetes mellitus (T2DM) to 16 demographically similar non-T2DM controls. Compared with controls, the T2DM group showed significantly less facilitation MEPs. Moreover, there was a positive correlation between iTBS-induced modulation of MEPs and verbal learning performance, suggesting a global decline in the efficacy of LTP-like plasticity in T2DM.

Trebbastoni et al. (2016) compared the impact of conventional 5 Hz rTMS to M1 on MEPs between 40 individuals with MCI and 20 older healthy adults. The participants received clinical, neurologic and neuropsychological assessments at baseline, and were followed for 4 years to monitor for conversion to AD. The authors reported a decrease in response to the rTMS protocol in individuals with MCI, which was correlated with time to conversion to AD. These results raise the possibility that rTMS could be a useful prognostic tool in the assessment of AD and related dementia.

The majority of cognitive aging-related studies using TBS and rTMS to probe the mechanisms of plasticity have focused on individuals with AD (Koch et al., 2012, 2014; Di Lorenzo et al., 2016; Fried et al., 2017). In the first of a series of studies, Koch et al. (2012) compared the response of 14 individuals with moderate AD and 14 older healthy adults to three different TBS protocols targeting M1: iTBS, cTBS, and coTBS (a facilitatory variant of cTBS combining continuous delivery of 300 pulses with a 1-min contraction of the first and second digits). In comparison to controls, individuals with AD showed a decreased response to iTBS and coTBS, but not cTBS, suggesting impaired LTP and spared LTD in AD. Similar to the findings in T2DM, the reduced response to iTBS was correlated with decreased performance on the delayed recall of the Rey's figure copy visuospatial task.

A subsequent study by the same group (Koch et al., 2014) replicated the decreased M1 iTBS response in 20 individuals with moderate AD. Moreover, LTP-like plasticity was recovered after a 4-week treatment with the dopamine agonist rotigotine, and was accompanied by an improvement in global cognition and executive function. These data indicate implicate dopaminergic pathways in the reestablishment of LTP-like plasticity, thus providing a potential therapeutic target.

Di Lorenzo et al. (2016) also evaluated iTBS and cTBS to M1 in 54 individuals with AD and 24 demographically similar older adults, confirming the prior findings of abnormal iTBS response and normal cTBS response in AD (Koch et al., 2012, 2014). Further, the authors found that iTBS not only failed to facilitate MEPs in AD, rather it led to the suppression of MEP responses similar to cTBS. An 18-month follow-up assessment demonstrated that the extent of the impaired response to iTBS was associated with more severe cognitive decline (Di Lorenzo et al., 2016).

While the aforementioned studies above suggest a potential usefulness in using TBS and conventional rTMS to assess the mechanisms of plasticity in older adults with and without cognitive impairment, a number of studies in young and older adults have highlighted the challenge posed by considerable inter- and intra-individual variability in the response to these protocols (Maeda et al., 2000; Vernet et al., 2014; Vallence et al., 2015; Fried et al., 2017; Schilberg et al., 2017). For example, Hamada et al. (2013) observed that only 25% of their 56 participants demonstrated the “expected” response to iTBS and cTBS (i.e., facilitation and suppression of MEPs, respectively). This variability was not related to age, gender, testing time, or difference in MT or amplitude of baseline responses. Interestingly, by altering the coil orientation (and thus, manipulating the direction of the resultant induced current in the brain), they found that at least 50% of this variability could be explained by individual differences in the latency of MEPs resulting from direct vs. indirect activation of layer-V pyramidal cells (Maeda et al., 2000).

Furthermore, Cheeran et al. (2008) demonstrated variability in the response to iTBS and cTBS could be partly explained by a commonly observed polymorphism of the brain-derived neurotrophic (BDNF) gene. The presence of a met allele in the BDNF gene has also been associated with higher test-retest variability in response to iTBS in older adults, including those with T2DM or AD (Fried et al., 2017). In that same study, Fried et al. (2017) examined the reliability of iTBS to M1 in 36 adults, including 9 with probable mild-to-moderate AD, 15 with T2DM and 12 older healthy adults. iTBS was at best associated with low reproducibility (Cronbach's α ≤ 0.50) in the older healthy adults and moderate to high reproducibility in individuals with AD (Cronbach's α 0.53 ≥ 0.81). The authors hypothesized that greater reproducibility in the clinical population could be linked to a neurobiological “rigidity” in the mechanisms that support LTP-like plasticity induction. While the reproducibility of iTBS has been evaluated in older adults, such data is not available for cTBS and rTMS. Additional contributing factors implicated with the variability of iTBS outcomes in older adults were the between-session time, rMT and baseline MEP amplitudes (Fried et al., 2017). Responses were more variable if the sessions were conducted within 7 days, possibly as a result of subtle longer-lasting changes in the expression of GABAergic precursors (Trippe et al., 2009). Echoing a point first raised in section Single-Pulse TMS, setting the intensity of subsequent stimulation based on a set level percentage of rMT may introducing additional variance given age-related changes in the input-output curve of TMS (Pitcher et al., 2003) and the relationships between changes in RMT, baseline MEP amplitude, and the impact of iTBS (Fried et al., 2017).

In summary, the potential usefulness of TBS and conventional rTMS to measure the mechanisms of plasticity in older adults across the spectrum of cognitive aging must be considered in context of the current limitations of high inter- and intra-individual variability. However, at least for AD (which comprise the majority of the findings to date), consistent results across studies coupled with higher reproducibility suggest that these measures are more stable, and therefore, more useful in this population. Furthermore, the associations between modulation of MEPs and disease progression, symptom severity, and response to treatment with cognitive behavior point to their clinical relevance. The insights offered by studying variability and its causes in older clinical populations are critical to the widespread implementation of these protocols. Thus, future studies should aim to replicate those findings with larger samples and investigate the reproducibility of cTBS and rTMS in older adults, with and without cognitive impairment.

### Utility of the motor cortex for TMS-based assessments of neurophysiology and neuroplasticity in cognitive aging and impairment

The vast majority of TMS based studies in cognitive aging described in this paper, and indeed in the field overall, are neurophysiological assessments probing the function of the motor cortex. Much of this has until recently been out of necessity, since the output of the cortico-spinal pathway provides the only objective response to a suprathreshold TMS pulse (i.e., a MEP). While improved technology and methods of data cleaning and analysis have expanded the potential of concurrent use of TMS and electroencephalogram (TMS-EEG) for assessing physiology in non-motor brain regions (Pascual-Leone and Taylor, 2011), the motor cortex remains the most well-characterized region and MEPs represent the gold standard for measuring the neurophysiological response to TMS. There is no doubt that restricting assessments to the motor cortex present a limitation to understanding the complex neurophysiology of the aging brain, especially as the motor cortex is rarely the target for disease-related pathologies, including those of AD.

However, there is an important benefit to conducting these assessments in a region, which is not directly affected by a particular disease. Take for example the case of AD, which is characterized by progressive spread of amyloid and tau depositions, cortical hypometabolism and atrophy. While the pathology of AD will eventually spread to involve the motor cortex, this is only at the latest stage of the disease (Braak and Braak, 1998), and the majority of studies to characterize or treat AD symptoms focus on patients at earlier stages of the disease, including preclinical populations where an intervention may be the most effective. Thus, while TMS based measurements in the motor cortex cannot inform on the function of brain regions directly affected by AD pathology, abnormal findings in motor cortex and be used to infer about the state of the brain overall rather than local pathology.

By comparison, if the measurements were performed in a region that was directly affected (such as association regions of frontal, parietal, or temporal lobes) it would be challenging to disentangle the contributions of local vs. global brain changes when making conclusions. Moreover, as TMS measurements are increasingly performed in non-motor brain regions, those findings, even in healthy cognitive aging, will still need to be explained and understood in the context of those same assessments performed in motor cortex (Gedankien et al., 2017).

### Therapeutic role of rTMS in cognitive aging and impairment

The rationale for the use of rTMS therapeutically is based in part on studies in elderly individuals that have reported altered patterns of activation in the prefrontal cortex associated with deficits in episodic memory (Kim, 2011). Moreover, participants receiving daily high-frequency rTMS to the left DLPFC for depression performed better in working memory, executive function, objective memory, and fine motor speed after treatment (Martis et al., 2003). Given the impairments observed in the retrieval and encoding of episodic memory exhibited in individuals with cognitive impairment (Nyberg et al., 1996), researchers are increasingly directing rTMS to cortical regions that operate in the execution of these functions. Episodic memory relies on activation of several areas, such as the medial temporal lobe, medial prefrontal cortex, and posterior parietal cortex (Wang et al., 2006). Among these, only the posterior parietal cortex is easily accessible to TMS. In addition, neuroimaging studies have demonstrated altered patterns of functional activity in the right DLPFC and hippocampus are related to memory decline in individuals with MCI and AD (Maillet and Rajah, 2013).

Single-session studies have been useful in contributing to knowledge of the consequences of the altered neurophysiology in the production of cognitive output in the aging brain and highlighted potential targets for multi-session studies. In older healthy adults, the performance of a prospective memory task can be augmented with iTBS to the Frontopolar cortex (Vidal-Piñeiro et al., 2014; Debarnot et al., 2015). In individuals with MCI, stimulation of the prefrontal cortex with high-frequency rTMS can improve processing speed, visuomotor coordination, executive function, and associative memory (Rektorova et al., 2005; Solé-Padullés et al., 2006). Alternatively, low-frequency rTMS to the right prefrontal cortex can improve recognition memory in individuals with MCI, and verbal and non-verbal memory in older healthy adults (Turriziani et al., 2012). Finally, iTBS to the right dorsolateral prefrontal cortex (R-DLPFC) can improve action and object naming in individuals with AD (Cotelli et al., 2008). Future studies should evaluate the efficacy of these interventions in multi-session studies with larger and better-characterized cohorts.

However, multi-session studies are more appropriate for drawing insights regarding therapeutic effectiveness. The studies discussed in this section are grouped as pertaining to a therapeutic role because they propose multi-session interventions involving rTMS and TBS.

Across all studies, the most common stimulation target has been the DLPFC. In older adults and individuals with MCI, this was the only therapeutic target studied to date. Kim et al. (2012) randomized 16 individuals to receive real 10 rTMS (13 2-s trains, inter-train interval of 15 s, 780 pulses/session) or sham to the left DLPFC for 5 days, and found improved inhibitory control, only in the real condition. Drumond Marra et al. (2015) randomized a group of 31 individuals with MCI to either undergo 10 sessions of real 10 Hz rTMS over the left DLPFC (5 s, with an inter-train interval of 25 s, 2,000 pulses/session) or sham. The authors found significant improvements in episodic memory post-stimulation and executive function only in the real group, retained at a follow-up assessment performed 1 month later.

In individuals with AD, two studies proposed the use of rTMS targeting the DLPFC in isolation. In the study by Cotelli et al. (2011). Ten individuals with moderate AD underwent 20 sessions of 20 Hz rTMS (50 trains, 2-s trains, inter-train interval of 28 s, 2,000 pulses/session) over 4 weeks. The authors found significant improvements in sentence comprehension at weeks 2 and 4, and at a follow-up performed 12 weeks post-intervention. Ahmed et al. (2012) randomized 45 individuals with mild-to-severe AD to one of 3 paradigms of DLPFC stimulation, delivered over 5 days: 20 Hz (20 5-s trains, inter-train interval of 25 s, 2,000 pulses/session); 1 Hz (2,000 pulses/session); and sham. The authors found that only individuals with mild to moderate AD showed any significant improvements. Participants in the 20 Hz group improved in global cognition, those in both the 20 and 1 Hz groups improved in instrumental daily activities, but the 20 Hz groups retained their improvements during a follow-up at 1 and 3 months post-stimulation.

A number of studies have used more than more target in individuals with AD. Zhao et al. (2017) recruited 30 participants and randomized them to 10 Hz rTMS (20 10-s trains, inter-train interval of 20 s, 2,000 pulses/session) targeting the following areas: bilateral DLPFC, bilateral parietal cortex, Broca's and Wernicke's area (superior temporal gyrus), or sham for 30 sessions over 6 weeks. The authors reported no between-group differences in any of the outcomes reported.

Five studies examined the use of rTMS targeting multiples areas combined with cognitive training in individuals with mild-to-moderate AD (Bentwich et al., 2011; Rabey et al., 2013; Rabey and Dobronevsky, 2016; Gandelman-Marton et al., 2017; Nguyen et al., 2017). All studies utilized cognitive training paradigms designed to engage the stimulated areas, with grammar, comprehension, action and object naming, and spatial memory and attention tasks.

Most of the studies (Bentwich et al., 2011; Rabey et al., 2013; Rabey and Dobronevsky, 2016; Gandelman-Marton et al., 2017; Nguyen et al., 2017) employed similar stimulation paradigms, consisting of 10 Hz rTMS (20–25 2-s trains, 1,200 pulses/session), targeting the following areas: bilateral DLPFC, bilateral parietal somatosensory association cortex, Broca's and Wernicke's area (superior temporal gyrus). Bentwich et al. (2011) recruited 8 participants, Rabey et al. (2013) recruited 15 participants, and Gandelman-Marton et al. (2017) recruited 8 participants, who underwent 54 sessions over 18 weeks. Rabey and Dobronevsky (2016) recruited 30 participants, who underwent 30 sessions over 6 weeks. All four studies demonstrated significant improvements in global cognition and activities of daily living both at 1.5 and 4.5 months following participation in the study, but Rabey et al. (2013) additionally demonstrated an increase in the participants' subjective perception of clinical change.

Nguyen et al. (2017) targeted the prefrontal and parietal cortices bilaterally, and Broca's and Wernicke's areas using 10 Hz rTMS (20 2-s trains, 400 pulses/session). Ten participants were recruited and underwent 25 sessions over 5 weeks. The authors found improvement in the overall score, and apathy and dependence sections of the Alzheimer's Disease assessment scale cognitive sub score (ADAS-Cog). Furthermore, a separate analysis limited to the five best responders revealed that their improvements remained at 6 months follow-up, while the improvements attained by the rest of the study sample did not.

With the possible exception of Rabey and Dobronevsky (2016), the results of the studies above are severely limited due to the small sample sizes and large inter-individual variability, resulting in low statistical power. In this scenario, it has been stipulated that effect sizes may be equally or even more useful than the simple consideration of statistical differences at estimating the clinical meaningfulness of trials (Ottenbacher, 1995; Musselman, 2007). For this reason, we calculated the effect sizes associated with the comparisons between interventions and control/comparison group from the studies above and considered that clinically meaningful changes to be *significant differences that were associated with an effect of at least a moderate size*. Effect sizes were computed using the Cohen's d (Cohen, 1988), calculated for within-group measures by computing the average standard deviation of both repeated measures (Cumming, 2012), and for between-group comparisons by dividing the difference between the means by the pooled standard deviation (Cohen, 1988) (Table 1). Effect sizes were interpreted based on published values: trivial effect (*d* < 0.2), small effect (*d* = 0.2–0.5), moderate effect (*d* = 0.5–0.8), and large effect (*d* > 0.8) (Cohen, 1988).

**Table 1:** Study characteristics from articles proposing rTMS as an intervention for cognitive improvement in individuals with cognitive aging and cognitive impairment.

**Study**	***n* Group status: age (mean ±*SD*)**	**Control/ Comparison**	**- Target area** **- Localization method**	**TMS parameters** **- Mode** **- Frequency** **- MT%**	**- Length of intervention** **- Sessions**	**TMS parameters** **- Trains** **- Duration** **- Interval** **- Total pulses**	**Cognitive domain**	**Neuropsychological test**	**Significant findings (treatment group)**	**Effect size (Cohen's *d*) between groups**	**Effect size (Cohen's *d*) within group**
Kim et al., 2012	16 OHA Active (8): 63.50 (±4.57) Sham (8): 62.75 (±5.50)	Sham site	L-DLPFC	- OFF /HF /rTMS - 10 Hz - 30%	- 1 week - 5 sessions	- 39 - 2s - NR - 780	1—Inhibitory control (RT) 2—ATT control	1 and 2–Modified Stroop task	↑ performance RT (incongruent trials) ↑ ATT control	DATA NOT SUFICIENT (not significant)	- RT congruent: 0.16 - RT incongruent: 0.51^**^
Drumond Marra et al., 2015	34 [aMCI (31) and naMCI (3)] Active (15): 65.1 (± 3.5) Sham (19): 65.2 (± 4.1)	Sham coil	- L-DLPFC - 5 cm anterior method	- HF/rTMS - 10 Hz - 110%	- 2 weeks - 10 sessions	- NR - 5 s - 25 s - 2,000	1—everyday MEM 2—EF	1 - RBMT, Logical memory II (delayed), WAIS III; 2 – TMTB and VF (animal naming).	↑ everyday MEM at 2 and 4 weeks ↑ EF (at 4 weeks)	- RBMT T1: 0.27 - RBMT T2: 0.57 - Logical memory II T1: −0.15 - Logical memory II—T2: −0.21 - Letter-number T1: 0.03 - Letter-number T2: 0.59 - TMTB T1: −0.34 - TMTB T2: 0.29 - Verbal fluency/animal T1: 0.08 - Verbal fluency/animal T2: −0.03	**WITHIN GROUPS (T0** × **T1)** - RBMT: 0.92^**^ - Logical memory II: −0.03^**^ - Letter-number: −0.20 - TMTB: −0.21^**^ - Verbal fluency/animal: −0.09^*^ **WITHIN GROUPS (T0** × **T2)** - RBMT: 1.16^**^ - Logical memory II: 0.56^**^ - Letter-number: 0.3 - TMTB: 0.12 - Verbal fluency/animal: 0.06^**^ **WITHIN GROUPS (T1 X T2)** - RBMT: 0.18 - Logical memory II: 0.57 - Letter-number: 0.52^**^ - TMTB: 0.29^**^ - Verbal fluency/ animal: 0.14
Cotelli et al., 2011	10 [moderate AD] Real-real (5): 71.2 (±6.1) Placebo-real (5): 74.4 (±3.8)	Placebo-real group: 2 weeks placebo + 2 week of real	- L-DLPFC - Template MRI guiding	- OFF/HF/rTMS - 20 Hz - 100%	- 4 weeks - 20 sessions	- 40 - 2 s - 28 s - 2,000	1—GC 2—MEM 3—EF 4—ADL and IADL; and 5—Language.	1—MMSE and Cognitive estimation test; 2 and 3 —picture-naming task 4—ADL and IADL task; 5—SC-BADA.	↑ Language (Persistent beneficial effects (along 2, 4, and 12 weeks) of rTMS in the real-real group on sentence comprehension in AD patients analyzed via SC-BADA)	**Variable: (2 weeks)/(4 weeks)/(12 weeks)** - MMSE: 0/−0.26/0.58 - ADL: −0.67/−0.67/−0.67 - IADL: −0.34/−0.34/−0.34 - Picture-naming task (objects): 0.55/0.71/1.05 - Picture-naming task (actions): 0.94/0.45/0.22 - Battery (Sentence comprehension): 1.54^**^/−0.25/0.35 - AAT (token test): 0.63/0.89/0.16 - AAT (Repetition): 0.13/0.8/0.54 - AAT (Writing): 0.03/0.02/0.03 - AAT (Naming): 0.11/0.28/0.73 - AAT (Comprehension): 0.2/0.12/0.82 - Serial curve position (primacy): −45/−0.66/−1.00 - Serial curve position (recency): 0.91^*^/0.86^*^/ 0.3^*^ - Serial curve position (first item): −0.66/−0.87/−0.62 - Cognitive estimation test (errors): 0.18/0.53/0.65 - Cognitive estimation test (bizarreness): 0.29/0.69/0.93	**Variable: (BASELINE** × **2 weeks)/(BASELINE** × **4 weeks)/(BASELINE** × **12 weeks)** - MMSE: −0.07/−0.26/0.07 - ADL: 0/0/0 - IADL: 0/0/0 - Picture-naming task (objects): −0.13/0.08/0.27 - Picture-naming task (actions): 0.82/0.56/0.62 - Battery (Sentence comprehension): 1.57^**^/0.92/1.54^**^ - AAT (token test): 0.22/0.68/0 - AAT (Repetition): −0.23/0.24/−0.04 - AAT (Writing): 0.41/0.61/0.53 - AAT (Naming): 0.06/0.55/1.31AAT (Comprehension): 1.08^*^/0.89/1.94 - Serial curve position (primacy): 0.31^*^/0/−0.1 - Serial curve position (recency): 0.44/0.8^*^/0.73^*^ - Serial curve position (first item): 0/−0.1/−0.9^*^ - Cognitive estimation test (errors): 0.54/0.65/0.48 - Cognitive estimation test (bizarreness): −0.27/0/0.4
Cotelli et al., 2011	10 [moderate AD] Real-real (5): 71.2 (±6.1) Placebo-real (5): 74.4 (±3.8)	Placebo-real group: 2 weeks placebo + 2 week of real	- L-DLPFC - Template MRI guiding	- OFF/HF/rTMS - 20 Hz - 100%	- 4 weeks - 20 sessions	- 40 - 2 s - 28 s - 2,000	1—GC 2—MEM 3—EF 4—ADL and IADL; and 5—Language.	1—MMSE and Cognitive estimation test; 2 and 3 —picture-naming task 4—ADL and IADL task; 5—SC-BADA.	↑ Language (Persistent beneficial effects (along 2, 4, and 12 weeks) of rTMS in the real-real group on sentence comprehension in AD patients analyzed via SC-BADA)	**Variable: (2 weeks)/(4 weeks)/(12 weeks)** - MMSE: 0/−0.26/0.58 - ADL: −0.67/−0.67/−0.67 - IADL: −0.34/−0.34/−0.34 - Picture-naming task (objects): 0.55/0.71/1.05 - Picture-naming task (actions): 0.94/0.45/0.22 - Battery (Sentence comprehension): 1.54^**^/−0.25/0.35 - AAT (token test): 0.63/0.89/0.16 - AAT (Repetition): 0.13/0.8/0.54 - AAT (Writing): 0.03/0.02/0.03 - AAT (Naming): 0.11/0.28/0.73 - AAT (Comprehension): 0.2/0.12/0.82 - Serial curve position (primacy): −45/−0.66/−1.00 - Serial curve position (recency): 0.91^*^/0.86^*^/ 0.3^*^ - Serial curve position (first item): −0.66/−0.87/−0.62 - Cognitive estimation test (errors): 0.18/0.53/0.65 - Cognitive estimation test (bizarreness): 0.29/0.69/0.93	**Variable: (BASELINE** × **2 weeks)/(BASELINE** × **4 weeks)/(BASELINE** × **12 weeks)** - MMSE: −0.07/−0.26/0.07 - ADL: 0/0/0 - IADL: 0/0/0 - Picture-naming task (objects): −0.13/0.08/0.27 - Picture-naming task (actions): 0.82/0.56/0.62 - Battery (Sentence comprehension): 1.57^**^/0.92/1.54^**^ - AAT (token test): 0.22/0.68/0 - AAT (Repetition): −0.23/0.24/−0.04 - AAT (Writing): 0.41/0.61/0.53 - AAT (Naming): 0.06/0.55/1.31AAT (Comprehension): 1.08^*^/0.89/1.94 - Serial curve position (primacy): 0.31^*^/0/−0.1 - Serial curve position (recency): 0.44/0.8^*^/0.73^*^ - Serial curve position (first item): 0/−0.1/−0.9^*^ - Cognitive estimation test (errors): 0.54/0.65/0.48 - Cognitive estimation test (bizarreness): −0.27/0/0.4
Ahmed et al., 2012	Total: 45 [mild to moderate and severe AD] Age: 68.4	Sham DLPFC	- R-DLPFC and L-DLPFC - 5 cm anterior method	- HF and LF/rTMS - 1–20 Hz - 90–100%	- 5 consecutive days - 5 sessions	- 20 - 5 s - 25 s - 2,000	1—GC; and 2—IADL	1—MMSE; and 2—IADL.	↑ GC and IADL in mild to moderate AD, not in severe (HF showed better improvement than LF) along 1 and 3 months.	**Legend: Variable: (after last session/1 month/3 months) MILD to MODERATE (20HZ** × **1 Hz)** - MMSE: 1.56/1.61/1.84 **SEVERE (20HZ** × **1 Hz)** MMSE: 0.42/1.48/1.15 **MILD to MODERATE (20HZ** × **SHAM)** - MMSE: 2.00/3.00/3.22 **SEVERE (20HZ** × **SHAM)** - MMSE: 1.3/1.67/2.03 **MILD to MODERATE (1HZ** × **SHAM)** - MMSE: 2.00/3.00/3.22 **SEVERE (1HZ** × **SHAM)** - MMSE: 1.3/1.67/2.03	**Legend: Variable: (BASELINE** × **AFTER)/(BASELINE** × **1 MONTH)/(BASELINE** × **3 MONTHS) 20Hz** - MILD to MODERATE MMSE: 1.01^**^/1.66^**^/2.00^**^ - SEVERE MMSE: 0.33/0/0.44 **1Hz:** - MILD to MODERATE MMSE: 0.05/0.30/0.34 - SEVERE MMSE: 0/−1.00/-0.68 **SHAM:** - MILD to MODERATE MMSE: 0.07/−0.17/−0.33 - SEVERE MMSE: 0.15/0/0
Bentwich et al., 2011	8 [mild to moderate AD] Age: 75.4 (±4.4)	No control	- R-DLPFC and L-DLPFC; BR-WE; R-pSAC and L-pSAC - Subject MRI guiding	- HF/rTMS/ COG - 10 Hz - 90–110%	- 18 weeks - 54 sessions	- 2 - 5 s - 30 s - 2,000	1—GC; and 2—IADL	1—ADAS-cog, CGIC, and MMSE; 2—ADAS-ADL	↑ ADAS-cog at 6 and 18 weeks (great improvement) ↑ ADAS-ADL and MMSE at 18 weeks ↑ CGIC (no statistical power)	No control group	**BASELINE** × **6 WEEKS** - ADAS-COG: 0.68^**^ - CGIC: −2 - MMSE: 0.62^**^ - ADAS-ADL: 0.33^**^ **BASELINE** × **4.5 months** - ADAS-COG: 0.85^**^ - CGIC: −4.57 - MMSE: −0.42 - ADAS-ADL: 0.09
Gandelman-Marton et al., 2017	8 (mild AD) Age: 75.5 (4.3)	No control	- Frontal, temporal, and parieto-occipital regions - Template MRI guiding	- ON/HF/rTMS/ COG - 10 Hz - 70–90%	- 18 weeks - 54 sessions (5x/week and 2x/week)	- 20 - 2 s - 40 s - 1,200	1—GC	1—ADAS-cog, CGIC, and MMSE;	↑ MMSE 6 weeks ↑ ADAS-cog (6 and 4.5 months	No control group	**BASELINE** × **6 WEEKS** - ADAS-COG: 0.68^**^ - MMSE: 0.65^**^ **BASELINE** × **4.5 months** - ADAS-COG: 0.84^**^ - MMSE: −0.37
Nguyen et al., 2017	10 (AD) Age: 73 (7.2)	No control	- 6 brain areas (R-DLPFC, L-DLPFC, R-PC, L-PC, BR-WE) - MRI guided	- HF/rTMS/ COG - 10 Hz - 100%	- 5 weeks - 25 sessions (5 days a week with a 6 months follow up)	- 20 - 2 s - 40 s - NR	- 1—GC - 2—MEM - 3—ATT and PS - 4—Language - 5—Visuospatial	- 1—MMSE, ADAS-cog; FAB - 2—Dubois score; - 3—Stroop color; - 4—Word recognition and word recall scores 5	↑ ADAS-cog at 5 weeks (scores returned after follow up, but not in best responders) ↑ Improvement of the performance on all task (test-retest effect and an effective learning) - After correction only task from parietal and languages were significant	- ADAS-cog (Best scores × Worst scores): 1.53^**^	**Variable: (Baseline** × **6 weeks)/(Baseline** × **6 months)** - ADAS-cog: 1.03^**^/0^**^ - ADAS-cog recognition: 0.45/0.89 - ADAS-cog word recall: 0.2/0 - MMSE score: 0.28/−0.31 - MMSE language score: 0.57/0.5 - Dubois score: −0.8/−0.74 - FAB score: 0.15/−0.07 - Stroop color: −0.45/0.17 **Variable (Cognitive task): (Baseline** × **6 weeks)** - R-DLPFC (action naming): 7.3 - R-DLPFC (subject naming): 6.4 - R- DLPFC (word recall): 7.3 - L-DLPFC (action naming): 8.06 - L-DLPFC (subject naming): 7.52 - L- DLPFC (word recall): 6.10 - R-PC (red rectangle recognition): 8.9^**^ - R-PC (blue rectangle recognition): 8.99^**^ - R-PC (letter recognition): 9.14^**^ - Broca (sentence similarity): 7.19 - Broca (right/wrong words): 9.52 - Wernicke (words/pseudowords): 11.37^**^ - Wernicke (categories): 15.21^**^
Zhao et al., 2017	30 [AD mild group and moderate group)] Age: 68.4	Non rTMS sham group	Parietal P3/P4 and posterior temporal T5/T6	- HF/rTMS - 20 Hz - NR	- 6 weeks - 30 sessions (1 session /day and 5 days/week)	- 20 - 10 s - 20 s - 2,000	- 1—GC - 2—MEM	- 1—ADAS-COG, MMSE, MoCA - 2—WHO-UCLA AVLT	↑ ADAS-cog, MMSE and WHO-UCLA AVLT score after treatment and 6 weeks along	**ALL (6 weeks/12 weeks)** - ADAS-cog: 0.62/0.57 - MMSE: 0.28/0.29 - MoCA: 0.07/0.07 - WHO-UCLA-AVLT: 0/0.11 **MILD (6 weeks/12 weeks)** - ADAS-cog: 0.79/0.76 - MMSE: 0.23/0.39 - MoCA: 0.03/0.27 - WHO-UCLA-AVLT: 0.19/0.53 **MODERATE (6 weeks/12 weeks)** - ADAS-cog: 0.51/0.60 - MMSE: 0/0.06 - MoCA: −0.08/-0.05 - WHO-UCLA-AVLT: −0.09/−0.16	**ALL (Baseline** × **Post 6 weeks)/(Baseline** × **12 weeks)** - ADAS-cog: 0.72^**^/0.90^**^ - MMSE: 0.64^*^/0.89^**^ - MoCA: 0.36/0.96 - WHO-UCLA-AVLT: 0.42/0.73^**^ **MILD (Baseline** × **Post 6 weeks)/(Baseline** × **12 weeks)** - ADAS-cog: 0.87^**^/1.07^**^ - MMSE: 0.48/1.24^**^ - MoCA: 0.53/0.86^**^ - WHO-UCLA-AVLT: 0.38/1.01^**^ **MODERATE (Baseline** × **Post 6 weeks)/(Baseline** × **12 weeks)** - ADAS-cog: 0.53/0.74 - MMSE: 0.41/0.73 - MoCA: 0.22/0.44 - WHO-UCLA-AVLT: 0.61/0.73 **COGNITIVE DOMAIN 6** × **12 weeks: (All/Mild/Moderate)** - MEMORY: 0.19^**^/0.12^**^/0.31 - LANGUAGE: 0.14^**^/0.11^**^/0.13 - EXECUTIVE FUNCTION: −0.2/0^**^/−0.7
Rabey and Dobronevsky, 2016	Total: 30 [mild to moderate AD] Treat (7): 72.6 (± 8.9) Sham (8): 75.4 (± 9.07)	No control	L-IFG (Broca); L-STG (Wernicke); L-DLPFC; R-DLPFC; R-pSAC and L-pSAC	- ON/HF/rTMS/ COG - 10 Hz - 90–110%	- 6 weeks - 30 sessions (1-h daily sessions, 5 days per week)	- 20 > 5 - 2 s > 2 s - NR - 1,200 > 100 = 1,300	1—GC	1—ADAS-cog and MMSE	↑ ADAS-cog and MMSE along 6 weeks	No control group	- ADAS-cog: 0.32^**^ - MMSE: 0.62^**^
Rabey et al., 2013	Total: 15 [mild to moderate AD] Treat (7): 72.6 (± 8.9) Sham (8): 75.4 (± 9.07)	Sham coil	R-DLPFC and L-DLPFC; BR-WE; R-pSAC and L-pSAC - Subject MRI guiding	- HF/rTMS/ COG - 10 Hz - 90–110%	–	- 20 - 2 s - NR - 1,200	1—GC	1—ADAS-cog and CGIC.	↑ ADAS-cog and CGIC along 6 and 18 weeks	Not enough information	Not enough information

*n, number of subjects; SD, standard deviation; TMS, Transcranial Magnetic Stimulation; rTMS, repetitive TMS; rtMS-COG, rTMS plus cognitive training; MT%, Motor Threshold percentage; OHA, Older Healthy Adults; AD, Alzheimer's Disease; MCI, Mild Cognitive Impairment; aMCI, amnestic MCI; naMCI – nonamnestic MC; T0, baseline; T1, first assessment after treatment; T2, second assessment after treatment; L-DLPFC, left dorsolateral prefrontal cortex; R-DLPFC, right dorsolateral prefrontal cortex; L-IPL, left inferior parietal lobe; PFC, Prefrontal cortex; R-IFG, right inferior frontal gyrus; R-STG, right superior temporal gyrus; BR-WE, Broca and Wernicke area; L-pSAC, left parietal somatosensory association cortex; R-pSAC, right parietal somatosensory association cortex; L-PC, left parietal cortex; R-PC, right parietal cortex; HF, High Frequency; LF, Low frequency; ON, online stimulation; OFF, offline stimulation; RT, reaction time; GC, global cognition EF, executive function; MEM, memory; ATT, attention; IADL, instrumental activities of daily living; VSP, visuospatial; ADL, activities of daily living; NAM, naming; PS – processing speed; MMSE, Mini-Mental State Examination; IADL, Instrumental Activities of Daily Living;; AAT, Aachener Aphasic Test; ADAS-cog, Alzheimer Disease Assessment Scale-Cognitive; ADAS-ADL, Alzheimer Disease Assessment Scale-ADL; CGIC, Clinical Global Impression of Change scale; NPI, Neuropsychiatric Inventory; TMT, Trail Making Test; ↑, improvement of; NA, Not applied; SC-BADA, Battery for Analysis of Aphasic Deficits; WHO-UCLA-AVLT, World Health Organization-University of California-Los Angeles Auditory Verbal Learning Test; MoCA, Montreal Cognitive Assessment; MIS, Memory Impairment Screen; FCRT, Free and Cued Recall Test; AVLT, Auditory Verbal Learning Test; RBMT, Rivermead Behavioral Memory Test; RT, reaction time; VFT, Verbal fluency test; STR, stroop test; RCFT-R, Rey Complex Figure Test and Recognition Trial; MRI, Magnetic Resonance Imaging; NR, not reported*;

** *, significant finding (p < 0.05)*;

* *, trend to significance (0.05 < p < 0.1)*.

Many significant within-group improvements were associated with at least moderate effect sizes. Regarding global cognition measured by the Mini Mental Status Exam (MMSE), multi-target 10 Hz rTMS combined with cognitive training (Bentwich et al., 2011; Rabey and Dobronevsky, 2016; Gandelman-Marton et al., 2017) was associated with moderate-to-high effect (*d* = 1.01–2.00, *d* = 0.62, and *d* = 0.64–1.24, respectively). In addition, 10 Hz rTMS in isolation (Zhao et al., 2017) was associated with a moderate effect (*d* = 0.65). Furthermore, multi-target 10 Hz rTMS combined with cognitive training (Bentwich et al., 2011; Gandelman-Marton et al., 2017; Nguyen et al., 2017) was also associated moderate-to-high effect for global cognition measured by ADAS-cog was also associated with a moderate to large effect (*d* = 0.68, *d* = 0.68, *d* = 1.03). A moderate-to-high effect was also associated with 10 Hz rTMS in isolation (Zhao et al., 2017; *d* = 0.72–1.07).

Additional clinically relevant effects were associated with 10 Hz rTMS in isolation were improved memory with 10 Hz rTMS (Drumond Marra et al., 2015; *d* = 0.56-1.16), verbal learning (Zhao et al., 2017; *d* = 0.73–1.01), and inhibitory control (Kim et al., 2012; *d* = 0.51). With 20 Hz rTMS in isolation, large effects were observed with improvements in sentence comprehension (Cotelli et al., 2011) (*d* = 1.54–1.57). All of the remaining within-group significant differences were associated with a lower than moderate effect.

Six studies (Bentwich et al., 2011; Cotelli et al., 2011; Kim et al., 2012; Drumond Marra et al., 2015; Rabey and Dobronevsky, 2016; Nguyen et al., 2017; Zhao et al., 2017) had a control/comparison group and presented their data with sufficient detail to allow for between-group Cohen's *d*. The improvements in the language domain associated with 20 Hz rTMS to the DLPFC was associated with a large effect (*d* = 1.54) (Cotelli et al., 2011). In addition, the improvement associated with global cognition associated with the multi-target 10 Hz rTMS combined with cognitive training was also large (*d* = 1.53) (Nguyen et al., 2017). All of the remaining significant between-group differences were associated with lower than moderate effect.

In summary, the above evidence suggests that after an intervention consisting of multi-target high-frequency rTMS combined with cognitive training as described above, individuals may present with clinically meaningful improvements in global cognition. This finding was consistent and replicated by four different studies. In addition, individuals participating in a high-frequency rTMS to the DLPFC can also achieve improvements in global cognition, but this finding was less consistent. Additionally, these individuals may exhibit clinically meaningful improvements in in memory, verbal learning, inhibitory control and sentence comprehension.

One important caveat is that the clinical meaningfulness of high-frequency rTMS, with or without cognitive training was limited when compared to a control condition. In this scenario, it may be possible to improve in global cognition when participating in multi-target rTMS with cognitive training and sentence comprehension when undergoing high-frequency rTMS in isolation. One limitation from the studies above is that none employed a comparison group; therefore, it is unclear if these results would persist in the comparison to an actual intervention. Future studies should employ active comparisons in their study design to gain a better understanding of the efficacy of these techniques.

## Challenges posed and opportunities offered by the variability of TMS

As discussed in many of the individual sections above, inter- and intra-individual variability remains a challenge to the widespread use of TMS as a neurophysiological probe and a neuromodulatory intervention. While this problem is not unique to the study of cognitive aging, there are specific considerations that must be addressed. For starters, much of our knowledge of how TMS interacts with the brain comes from the study of younger, relatively healthy individuals. Thus, it may be necessary to develop age-specific normative values if these TMS assessments are to be used to distinguishing normal from pathological cognitive aging. Furthermore, TMS measures that are already highly variable across subjects within a given group are likely to have poor diagnostic utility in older populations, especially given the highly individualized trajectories of cognitive aging and the large heterogeneity of age-related pathologies.

Similarly, the problem of intra-individual variability, or reproducibility, of certain TMS measures is a direct limitation to their use as prognostic tools to track individual decline or response to therapeutic interventions. Fortunately, these problems are increasingly recognized within the TMS scientific community leading to several recent reviews (Gonsalvez et al., 2017), meta-analyses (Wischnewski and Schutter, 2015; Chung et al., 2016), and direct investigations into the magnitude and sources of inter- and intra-individual variability (Cheeran et al., 2008; Chang et al., 2016; Schilberg et al., 2017), including recent analyses of older, clinical populations (Christie et al., 2007; Fried et al., 2017).

While variability is often thought of purely in terms of the limitations it confers, there may be opportunities to exploit inter-individual variability to gain a deeper understanding into the neurobiology of cognitive aging. One such example is in the study of T2DM, a major contributor to pathological cognitive aging in its own right as well as a risk factor for dementia (Arvanitakis et al., 2004). As mentioned in section TBS and rTMS to Assess Plasticity in Cognitive Aging and Impairment, Fried et al. (2016) compared older diabetics without cognitive complaints to non-T2DM controls. Not only did the T2DM show a decreased response to iTBS, there was a strong association in the T2DM group between the modulation of MEP and verbal learning performance. Thus, the variation in response to iTBS within the T2DM group was actually informative as to more general disruption in LTP-like plasticity that also impacted learning and memory. Similarly, while several studies have used motor thresholds and other TMS measures as evidence of hyperexcitability in AD (Ferreri et al., 2003; Di Lazzaro et al., 2004; Koliatsos et al., 2006), the variation in rMT may be itself predictive of cognitive dysfunction. Finally, in order to fully leverage the sources of variability to better inform on the neurobiology of cognitive aging, it may be necessary to look beyond straightforward analysis of variance or simple linear regression techniques when analyzing data.

## Transcranial direct current stimulation (tDCS)

While TMS is a method that functions through direct generation of action potentials in cortical tissue using electromagnetic induction, tDCS is an alternate NIBS method that achieves neuromodulation of both cortical and subcortical tissue through sub-threshold alteration of resting membrane potentials using direct electrical stimulation. tDCS is safe to use and has shown potential for enhancing cognitive processes and intervening on disease states (e.g., depression, chronic pain, recovery after stroke) (Nitsche and Paulus, 2000; Fregni et al., 2006; Reis et al., 2009; Medeiros et al., 2012; Ahn et al., 2017). In tDCS, a weak electrical current (typically 1–2 mA) is delivered to underlying brain tissue through electrodes placed over the scalp (Nitsche and Paulus, 2000; Nitsche et al., 2008). Saline-soaked sponges, or carbon loaded rubber pads with conductive gel are used as electrodes for direct current to flow between the anode (positive polarity) and cathode (negative polarity) electrode. Current flows between the electrodes may alter the sub-threshold resting membrane potential of neurons, resulting in either neural excitation or inhibition of cortical and subcortical tissues (Bolzoni et al., 2013; Stagg et al., 2013). Unlike TMS, tDCS has the potential to impact underlying subcortical tissue either directly or indirectly. Computational modeling (Sadleir et al., 2010; Shahid et al., 2014; Indahlastari et al., 2016) and intracranial recording data (Opitz et al., 2016; Huang et al., 2017) have demonstrated that electrical current passing between tDCS electrodes was found in both intervening cortical and subcortical tissue and thus may directly stimulate these brain regions (i.e., direct effect). In addition, effects on subcortical tissue can also be achieved indirectly through complex connectivity of brain network within the stimulated and unstimulated cortical and subcortical brain regions (Weber et al., 2014).

The parameters used in tDCS are assumed to dictate whether the neural response to stimulation under and between the anode or cathode electrode is excitatory or inhibitory. These parameters are consisted of stimulation duration and frequency, current intensity, as well as electrode size and location (Prehn and Flöel, 2015). Studies have shown that stimulation intensity of 1 mA results in depolarization of neurons under the anode and hyperpolarization under the cathode electrode (Nitsche and Paulus, 2000). However, stimulation at 2 mA has previously been shown to elicit net excitatory response under both the anode and cathode electrodes (Batsikadze et al., 2013). In addition, the effects of tDCS can outlast the period of stimulation from minutes to hours post-stimulation depending on the duration of stimulation (Bindman et al., 1964; Monte-Silva et al., 2013). The size of stimulation can be achieved by using different types of electrodes. Conventional tDCS offers a broader stimulation area via two large (e.g., 35 cm^2^) rectangular electrodes (one anode, one cathode). High-definition tDCS (HD-tDCS) can provide more focal stimulation regions by using smaller disc electrodes (1 cm diameter) in a 4 × 1 ring configuration (e.g., anode in the center surrounded by four cathode electrodes) (Kuo et al., 2013). Therefore, the choice of conventional vs. HD-tDCS is based on the location of desired target brain regions. Electrode montages used in tDCS were selected such that the intended stimulation regions would receive a sufficient current dose. Studies of cognition in tDCS typically follow a functional targeting approach that specifically aims to stimulate underlying brain regions implicated in the cognitive abilities of interest for a given study. For example, the most common intended stimulation area for cognitive aging is the frontal lobe in an effort to impact executive functions such as working memory and error awareness (Nissim et al., 2017). In addition, decline in frontal structures and function are a hallmark of the cognitive aging process and represent one of the brain areas most impacted by advanced age (Lemaitre et al., 2012)—supporting the frontal lobes as a target for tDCS intervention. In AD population, the temporoparietal and temporal regions were chosen as the stimulation target areas since impairments in these regions have been correlated with impaired verbal and visual recognition observed in AD patients (Ferrucci et al., 2008).

Studies in both humans and animals have suggested that the acute effects (during stimulation) vs. the after-effects (post stimulation) are relying on different mechanisms of action (Liebetanz et al., 2002; McLaren et al., 2017). While the exact mechanisms of action are not well-understood, studies have shown that sodium and potassium channels are necessary for the acute effects of stimulation, whereas the after-effects of stimulation are NMDA receptor, GABA/Glutamate, and calcium channel dependent (Stagg et al., 2009a). The mechanisms of action that produce the after-effects of stimulation are particularly important when we consider cognitive aging and age-related cognitive decline. As previously discussed, certain aspects of cognition start to decline with aging (e.g., working memory, attention). The ability to modulate the excitatory response of brain tissues for longer periods of time can potentially enhance LTP, which is the basis of learning and memory. LTP indicates a persistent synaptic strengthening, providing the capacity of modifying neural connectivity that can support signal transmission between neurons (Bliss and Lomo, 1973; Barnes, 2003; Monte-Silva et al., 2013). By altering LTP and neuroplasticity in older adults, tDCS shows promise to remediate cognitive aging and age-related diseases (Nitsche and Paulus, 2000; Nitsche et al., 2008).

### tDCS to remediate cognitive aging in healthy older adults

Prior research in healthy older adults shows promise for the application of tDCS to remediate age-related cognitive decline and enhance cognition. Stephens and Berryhill (2016) examined tDCS paired with working memory training in 90 healthy older adults. Participants were randomly assigned to receive sham, 1 mA, or 2 mA of stimulation for 15 min while completing five sessions of working memory training. The anode electrode was placed over the right DLPFC (F4), and the cathode was placed over the contralateral cheek. Assessments were given pre- and post-intervention and all participants showed improvements on the trained verbal and visual working memory tasks. The group receiving 2 mA of stimulation showed a significant increase in far transfer benefits (processing speed, cognitive flexibility, arithmetic) at 1 month after intervention. This observation suggested that cognitive training paired with 2 mA of tDCS might induce overall improvements to daily life activities in older adults.

Park et al. (2013) assessed duration of tDCS effects combined with cognitive training on working memory in healthy older adults. Forty older adults were randomly assigned to sham or active tDCS during 10 sessions (over 2-weeks, 5 days/per week) of computer-assisted cognitive training (30 min a day). tDCS current was delivered over the bilateral prefrontal cortex (F3/F4) at 2 mA for 30 min in the active group. Using two stimulators, two anodes were placed over F3 and F4 and two cathodes were placed on the non-dominant arm. The sham group had an identical montage of electrodes with the stimulation device powered off after 30 s. Neuropsychological assessments were performed at baseline and 1-month post intervention. Significant improvements in digit span forward tests were seen in active vs. sham groups. The verbal working memory accuracy improvement was maintained for 28 days post intervention. These results suggested that tDCS might enhance cognitive training outcomes on cognitive functions in healthy older adults that lasted beyond the stimulation period.

In a task-based fMRI study with tDCS, Meinzer et al. (2013) examined the effects of improved language function induced by tDCS in 20 healthy older adults (age 60–76) and included healthy younger adults (aged 19–31) as controls. Active tDCS was delivered during a resting state scan followed by a semantic word generation task (1 mA for 17 min). The anode was placed over the left inferior frontal gyrus (IFG) (10–20 EEG system corresponding to the intersection of T3-F3 and F7-C3 and the midpoint between F7-F3) and the cathode on the contralateral supraorbital region. Reduced performance on semantic word-generation tasks in healthy older adults associated with enhanced task-related activity in bilateral IFG activation was found during sham stimulation. The active stimulation in older adults produced significantly higher performance comparable to the younger controls, and significantly reduced task-related hyperactivity in bilateral prefrontal cortex (PFC). Increased connectivity was also observed between the left IFG and the language related cortical areas during the resting state in active stimulation compared to sham. These results suggested that enhanced functional connectivity might be the basis for increasing neural efficiency marked by reduced activation with improved performance in task related cortical areas. Therefore, these results showed potentials of tDCS at enhancing cognitive processes in older adults with direct impact on underlying neural response patterns.

Harty et al. (2014) investigated the effects of tDCS on error awareness in healthy older adults by stimulating the right and left DLPFC during a sham-controlled, single-blind crossover trial. Participants were separated into four groups (24 healthy older adults per group). The study tested the influence of current polarity and electrode location (anode over F3 or F4, and cathode over Cz), on error monitoring. During tDCS application of 1 mA, participants performed a computerized test of error awareness (5 blocks, each 7.5 min and 1-min resting time within each block), a Go/No-go response inhibition task that required constant monitoring to detect errors. The group with anode placements over the right DLPFC (F4) was the only group to experience improved error detection during the task. This study suggested that the right DLPFC might have a larger causal role on error awareness compared to the left DLPFC.

### Therapeutic role of tDCS to remediate mild cognitive impairment and dementia in older adults

Previous studies have investigated the effects of tDCS in MCI populations and have found improvements in a variety of areas (e.g., face-name association memory, non-verbal recognition memory, attention) (Birba et al., 2017). In a double-blind, sham-controlled, within-subject study, Meinzer et al. (2015), applied tDCS at 1 mA for 20 min over the left ventral IFG (Brodmann areas 44/45) with the anode electrode placed on the left vIFG and the cathode on the right supraorbital region (Meinzer et al., 2012) in MCI participants. This study aimed to assess the effects of tDCS on cognition using a semantic word generation task. Multi-modal neuroimaging was acquired in a 3 Tesla MRI comparing resting state connectivity and task-related brain activity in active vs. sham tDCS groups. The active group improved significantly in word retrieval performance up to the same level as found in healthy older adults. Overall, findings from this study suggested that tDCS might improve cognitive performances in older adults with MCI by decreasing bilateral hyperactivity in PFC (Meinzer et al., 2015).

Yun et al. (2016) investigated the effects of repeated tDCS application on glucose metabolism and cognitive performance in MCI participants (*N* = 16). tDCS intensity of 2 mA was applied for 30 min, three times per week for 3 weeks in the active condition with the anode over left DLPFC (F3) and cathode over the right DLPFC (F4) for bilateral frontal stimulation. Using Positron emission tomography (PET), they found a significant increase in cerebral metabolic activity in the medial prefrontal cortex, precuneus, midtemporal regions, and the anterior cingulate cortices in the active group over sham. Multifactorial Memory Questionnaire (MMQ) was performed to assess participant's subjective memory functioning. MMQ scores and glucose metabolism were significantly improved in the active group over sham. Therefore, active tDCS can potentially change the regional brain metabolism as well as transient memory function in MCI participants (Steffener et al., 2009).

Studies using tDCS in AD participants have demonstrated positive effects on cognitive function when tDCS was applied during task execution (Hsu et al., 2015). Ferrucci et al. (2008) assessed the effects of tDCS applied over the temporoparietal areas in probable AD participants (*N* = 10). In this within-subject study, all participants received two active (reversed polarity) and one sham tDCS session over the temporoparietal areas. tDCS was delivered bilaterally via two pairs of electrodes. Each pair consisted of an electrode placed on the scalp (P3-T5 left side; P6-T4 right side) and another on the right deltoid muscle. For each session, recognition memory and visual attention were assessed pre-stimulation and 30-min post-stimulation. Accuracy for word recognition increased significantly with the anode placed over the temporoparietal region and reduced with the cathode over the temporoparietal region for the active tDCS sessions. There was no change in accuracy observed during the sham session (Ferrucci et al., 2008). This study demonstrated that stimulation over the temporoparietal areas might affect recognition memory in participants with AD. Further studies are necessary using repeated sessions in conjunction with therapeutic interventions (e.g., cognitive training) for treatments of cognitive decline in AD participants.

Boggio et al. (2012) examined the effects of tDCS on visual memory in AD participants in this within-subject study (*N* = 15). Cognitive functions were evaluated before and after the stimulation. tDCS was delivered at 2 mA for 30 min per day for five consecutive days. Bilateral stimulation was achieved using two anode electrodes placed over the temporal lobes (T3 and T4) and the cathode over the right deltoid muscle. All participants received active and sham stimulation, with sessions randomized by order and separated by an average of 71.1 days to avoid possible carry over effects. The results showed a main effect of active tDCS on enhancing visual memory performance over sham at baseline. However, there was no difference in general cognitive performance measured between active and sham (Boggio et al., 2012). This study demonstrated the therapeutic benefit of tDCS on visual memory in AD participants. Future studies aimed at optimizing intervention protocols can be explored to evaluate if specific task enhancements can be transferred to other cognitive domains.

Vascular dementia is the second highest prevalent form of dementia after AD. Vascular lesions typically result in cognitive slowing on a global scale including frontal lobe executive dysfunction and attention deficits, and local deficits at the lesion site (Hachinski et al., 2006; André et al., 2016). André et al. (2016) examined the effects of tDCS over the left DLPFC on visual short-term memory, executive function and working memory in participants with mild vascular dementia in a parallel-group design (*N* = 21; 13 active 8 sham). At-home tDCS at 2 mA for 20 min was performed over four consecutive days, with the anode placed over the left DLPFC (F3) and cathode over the contralateral supraorbital area (Fp2). In this study, participants completed cognitive assessments on the first day of stimulation, final day of stimulation, and 2-weeks later. The assessments were picture-naming task to assess visual short-term memory, the N-Back task to assess verbal working memory, and the Go/No-go task to assess executive control. Improvements were observed in both the active and sham groups. While the observed outcomes might be due to test-retest effects from repeated testing, the tasks could be considered as cognitive training, which is a promising tool for rehabilitation. Visual recall and reaction times on the N-Back and Go/No-go task were improved significantly in the active group over sham. This study provided compelling evidence for therapeutic potentials of tDCS combined with cognitive training or behavioral protocols aimed at preventing cognitive decline or rehabilitation of cognitive faculties.

## Summary and limitations of tDCS studies targeting cognitive aging and impairment in older adults

Collectively, clinical studies of tDCS in both healthy and impaired older adults have shown potential in improving cognition and functional independence in aging population. Table 2 summarizes a variety of stimulation parameters (electrode placement, duration, intensity, electrode size) of tDCS studies cognitive aging and impairment as mentioned in the previous sections. Cohen's *d* effect sizes were included in the table to demonstrate tDCS effects at the group level. Moderate to high effect sizes indicated that tDCS showed positive results in remediating cognitive aging and a variety of dementia-related cognitive impairments. Overall, the studies reported in the previous sections suggested that applications of 1–2 mA tDCS for at least 15 min showed moderate effects (Cohen's *d* > 0.5) to improve targeted cognitive functions in older adults. The majority of these tDCS studies (Ferrucci et al., 2008; Boggio et al., 2012; Park et al., 2013; Meinzer et al., 2015; André et al., 2016; Stephens and Berryhill, 2016; Yun et al., 2016) utilized electrode placements over the frontal lobe with memory as the most studied domain. However, it is difficult to identify a single parameter set with the highest potential for success due to the lack of common outcome measures and variation in the cognitive domains targeted across studies.

**Table 2:** Study characteristics from articles proposing tDCS as an intervention for cognitive improvement in individuals with cognitive aging and cognitive impairment.

**Study**	**Mean Age (year)/ sample size**	**Population**	**Intended stimulation region**	**Anode**	**Cathode**	**Electrode size (cm2)**	**Current (mA)**	**Duration (min)**	**Outcome measure**	**Cognitive domain**	**Effect size**
Stephens and Berryhill, 2016	68.2/*N* = 90	OHA	Frontal	F4	Contralateral cheek	35	2	15	Weekly Calendar Planning Activity; Road Law & Road Craft Test	Working Memory	0.56
Park et al., 2013	69.7/*N* = 40	OHA	Frontal	F3; F4	non-dominant arm	25	2	30	Digit span; 2-Back verbal working memory task	Working Memory	0.63
Meinzer et al., 2013	68.2/*N* = 20	OHA	Frontal	left vIFG	right supraorbital	35	1	17	Semantic word generation	Language Production	1.21
Harty et al., 2014	72.1/*N* = 48	OHA	Frontal	F4	Cz	35	1	37.5	Go/No-go response inhibition task	Error Awareness	0.49
Meinzer et al., 2015	67.4/*N* = 36	MCI	Frontal	left vIFG	right supraorbital	35	1	20	Semantic Word Retrieval	Memory - Non-verbal Recognition	1
Yun et al., 2016	74.8/*N* = 16	MCI	Frontal	F3	F4	25	2	30	MMQ-C (contentment)	Subjective Memory Complaints	1.11
Ferrucci et al., 2008	75.2/*N* = 10	AD	Temporoparietal	P6; T4	right deltoid muscle	32	1.5	25	Word recognition task accuracy	Memory—Verbal Recognition	2.8
Boggio et al., 2012	78.9/*N* = 15	AD	Temporal	T3; T4	right deltoid muscle	35	2	30	Visual recognition task performance	Memory—Visual Recognition	0.35
André et al., 2016	63–94^*^/*N* = 21	MVD	Frontal	F3	Fp2	35	2	20	Reaction time on 2-Back task	Working Memory	0.73

*N, number of subjects; tDCS, transcranial direct current stimulation; OHA, Older Healthy Adults; AD, Alzheimer's Disease; MCI, Mild Cognitive Impairment; MVD, Coronary Microvascular Disease; cm^2^, square centimeter; mA, miliampere; F3, left DLPFC; F4, right DLPFC; vIFG, Brodmann areas 44/45 (inferior frontal gyrus); P6, right side temporoparietal; T3, inferior gyri of temporal lobe; T4, fusiform gyrus of temporal lobe; Cz, vertex; Fp2, contralateral supraorbital area; MMQ, Multifactorial Memory Questionnaire*;

* *, age mean not reported*.

As the study of tDCS as an intervention for cognitive aging and dementia is a nascent field, the collective data presented in this paper perhaps best indicate the promise of this approach for future investigation and dose-response refinement. In addition, these studies converge in demonstrating stimulation of frontal cortices as a desirable target for intervention approaches in cognitive aging and dementia. At present, the first Phase III tDCS trial targeting remediation of cognitive aging was recently initiated and is underway (Woods et al., 2018). Trials of this type as well as further research integrating multimodal neuroimaging with tDCS in the study of cognitive aging and dementia will greatly improve our overall understanding of treatment efficacy and potentially provide a window to strategies for treatment optimization.

Factors impacting individual response to tDCS deserves further consideration and study. The clinical studies to date were mainly focused on treatment effects at group-level compared to individual effects. Individual variability in brain anatomy plays a critical role in the distribution and intensity of current flow delivered to the brain during tDCS, and thus may alter individual treatment effects (Minhas et al., 2012; Kessler et al., 2013; Woods et al., 2016). Finite element studies on current flow modeling using realistic head models have been used to predict the effects of inter-subject variability (Miranda et al., 2003; Datta et al., 2009; Opitz et al., 2013; Laakso et al., 2015). At present, while current flow modeling is available, individual assessments of current dosage *in-vivo* resulting from tDCS are limited (Kasinadhuni et al., 2017). Future studies are needed to investigate the cognitive effects from tDCS at the individual level and factors that may alter efficacy.

## TMS and tDCS use in cognitive aging: moving forward

NIBS such as TMS and tDCS are promising as methods for non-invasively probing cortical circuits *in-vivo*. Since its introduction in the 1980's, TMS has seen widespread use as a non-invasive means to assess cortico-motor neurophysiology and as a clinical intervention for certain chronic disorders, such as medical resistant major depression. Beyond these applications, evidence generated over the last years and summarized in the present review, highlights additional TMS application for aging such as the evaluation of the functional integrity of intracortical GABAergic, glutamatergic, and cholinergic circuits, the assessment of mechanisms of LTP and LTD-like plasticity, and novel potential therapeutic targets for stimulation. While tDCS literature spans less than 20 years, it is quickly becoming a common methodology for non-invasive brain stimulation in both research and clinical settings. As a safe and relatively painless method for modulating the excitability of brain tissue, tDCS has strong potential for application in a variety of aging-related disorders and conditions (Bikson et al., 2016; Szymkowicz et al., 2016; Ahn et al., 2017; Fazeli et al., 2017). Therefore, researchers and clinicians are exploring the use of these techniques to provide mechanistic insight into the pathophysiology of cognitive aging and cognitive impairment and as a potential means to characterize, or even slow or reverse cognitive decline.

The results of TMS and tDCS studies presented herein have been mixed, largely owing to the inherent challenge of studying a large heterogeneous population (older adults with and without cognitive impairment) without a consistent agreed-upon set of parameters for stimulation, or measurable and sensitive outcomes of cognition. These are necessary factors to move the field forward and improve both the characterization of individuals at baseline, and the responsiveness to therapeutic interventions. While several technique-specific sources of variability were identified and discussed in the previous sections (individual, genetic, methodological), collectively, group-level data from interventional applications of TMS and tDCS demonstrated clinically meaningful, positive behavior results, with the most promising and reliable parameters presented in Tables 1, 2. High-frequency rTMS was associated with improvements in global cognition, memory, verbal learning, inhibitory control and sentence comprehension. tDCS was associated with improvements in memory, working memory, language production, error awareness. However, one area of consistency across both rTMS and tDCS is the prevalence of studies targeting the frontal lobe in an attempt to enhance cognitive functions. This is perhaps not surprising, as the frontal lobe is one of the brain structures that changes most significantly due to advanced age, in the absence of neurodegeneration, and is associated with a myriad of cognitive abilities that decline with age.

Future studies aimed at addressing the limitations highlighted in the present study have the potential to further improve the efficacy of treatment effects at a group level. However, not all identified sources of variability can be constrained (for example, sources related with intra-individual differences in health status, anatomy or genetic make-up). For this reason, future studies should incorporate the characterization of these sources of variability in their samples and investigate the cognitive effects from TMS and tDCS further at individual level, rather than relegate this important point to the discussion section as a means to explain muddled or unexpected results. Further, the stimulation parameters included in this study are for using TMS and tDCS separately. Therefore, further consideration for parameters may be needed when applying multiple NIBS techniques simultaneously (Hamada et al., 2012). Although outside the scope of the current paper, other NIBS approaches using transcranial alternating current stimulation or transcranial random noise stimulation methods may also hold promise for cognitive aging and dementia. While the body of literature for these NIBS techniques is limited, these methods deserve additional study in this domain.

Furthermore, age-related changes in brain structure and resulting consequences for tDCS current flow and TMS-induced intracranial current are important factors that influence outcomes and require significant further study. In addition, understanding how age-related change in functional brain response impacts TMS and tDCS outcomes may provide important information for optimizing treatment gains in these studies. As we find better methods for understanding how the electrical current impacts non-motor tissue, we will have more ability to customize stimulation parameters such as current dosage and electrode placements, potentially creating paradigms that can be individualized.

In this context, neuroimaging (PET, MRI, EEG, etc.) can help. While TMS and tDCS can be applied in the absence of imaging, the integration of NIBS with multimodal human neuroimaging allows a more thorough investigation of structural and functional brain differences that are relevant for improving overall efficacy of TMS and tDCS outcomes. For instance, the use of fMRI to provide functional targeting for tDCS montage design (Woods et al., 2014) and rTMS targets (Drysdale et al., 2017) for intervention applications will serve to refine intervention protocols. Half of the interventional rTMS studies included in this review (Bentwich et al., 2011; Cotelli et al., 2011; Rabey et al., 2013; Gandelman-Marton et al., 2017; Nguyen et al., 2017) employed MRI-guided rTMS, providing support for the increased dissemination of the utility of integrating neuroimaging with NIBS (Table 1).

Furthermore, structural and functional imaging can be done prior to the stimulation to predict the responsiveness of individuals to NIBS interventions based on predicted or *in vivo* mapping of current flow, or individually select cortical targets based on task or resting-state activation, respectively (Weigand et al., 2017; Boes et al., 2018). With improved understanding of how current flow and intensity within specific brain regions impact behavioral outcomes, it may be possible to move toward an individualized approach for NIBS and parameter design for optimization of outcomes and patient selection. In addition, the increased use of neuronavigated systems to deliver TMS enables greater precision during stimulation.

With strong promise for a wide variety of applications in older adult populations, both TMS and tDCS represent NIBS techniques that may serve to address growing public health concerns for a rapidly growing elder population. Refining the application of these methods by taking the steps above will be important to pushing these methods further into clinical translational application in cognitive aging populations.

## Author contributions

JG-O, AW, AI participated in the study concept, manuscript preparation and revision. JR, DC, NN, SA, MM, AI participated in the data extraction and manuscript preparation. PF participated in the study concept and manuscript preparation.

### Conflict of interest statement

The authors declare that the research was conducted in the absence of any commercial or financial relationships that could be construed as a potential conflict of interest.
